# Early and short-term use of proprotein convertase anti-subtilisin–kexin type 9 inhibitors on coronary plaque stability in acute coronary syndrome

**DOI:** 10.1093/ehjopen/oeae055

**Published:** 2024-07-23

**Authors:** Hiroki Uehara, Takashi Kajiya, Masami Abe, Marohito Nakata, Shingo Hosogi, Shinichiro Ueda

**Affiliations:** Department of Cardiology, Urasoe General Hospital, 1-56-1/Maeda Urasoe City, Okinawa 9012102, Japan; Department of Clinical Research Education and Management, University of Ryukyus Graduate School of Medicine, 207 Uebaru Nishihara town, Okinawa 9030215, Japan; Department of Cardiology, Tenyoukai Central Hospital, Kagoshima, Japan; Department of Cardiology, Yuai Medical Center Hospital, Okinawa, Japan; Department of Cardiology, Naha City Hospital, Okinawa, Japan; Department of Cardiology, Hosogi hospital, Kochi, Japan; Department of Clinical Research Education and Management, University of Ryukyus Graduate School of Medicine, 207 Uebaru Nishihara town, Okinawa 9030215, Japan

**Keywords:** PCSK9 inhibitor, Acute coronary syndrome, LDL-cholesterol, Statin, Optical coherence tomography

## Abstract

**Aims:**

Proprotein convertase anti-subtilisin–kexin type 9 inhibitors (PCSK9Is) improve plaque volume and composition and reduce major adverse coronary events in chronic coronary artery disease. We evaluated the effects of the short-term use of PCSK9Is on coronary plaque stability in patients with acute coronary syndrome (ACS) using optical coherence tomography (OCT).

**Methods and results:**

This is a multicentre, open-label randomized controlled trial. The enrolled 80 subjects met the inclusion criteria. Of these, 52 patients (age 60 ± 11 years, 38 men, 14 women) with ST-elevated ACS had undergone successful primary percutaneous coronary intervention with LDL-cholesterol (LDL-C) levels > 70 mg/dL while receiving high-intensity statins. Participants were randomly assigned to the PCSK9I group (evolocumab 420 mg for 3 months, *n* = 29) or the standard of care (SoC) group (*n* = 23). Optical coherence tomography was performed at baseline (BL) and 3 and 9 months after randomization to assess lipid-rich plaques in non-culprit lesions. The change in the minimum fibrous cap thickness (MFCT) from BL to 9 months was the primary endpoint. The percentage change in LDL-C levels from BL to 3 months was significantly greater in the PCSK9I group (−67.8 ± 21.5% in the PCSK9I group vs. −16.3 ± 21.8% in the SoC group; *P* < 0.0001), and the difference between the two groups disappeared from BL to 9 months (−20.0 ± 37.8% in the PCSK9I group vs. −6.7 ± 34.2% in the SoC group; *P* = 0.20). The changes in MFCT from BL to 9 months were significantly greater in the PCSK9I group, even after PCSK9I discontinuation {100 μm [interquartile range (IQR): 45–180 μm] vs. 50 μm [IQR: 0–110 μm]; *P* = 0.032}.

**Conclusion:**

Combination treatment with PCSK9Is and statins resulted in more marked plaque stabilization after ACS than SoC alone, and this effect persisted for 6 months after PCSK9I discontinuation.

**Registration:**

Adage-Joto study, UMIN ID No. 26516.

## Graphical Abstract

**Graphical Abstract:**
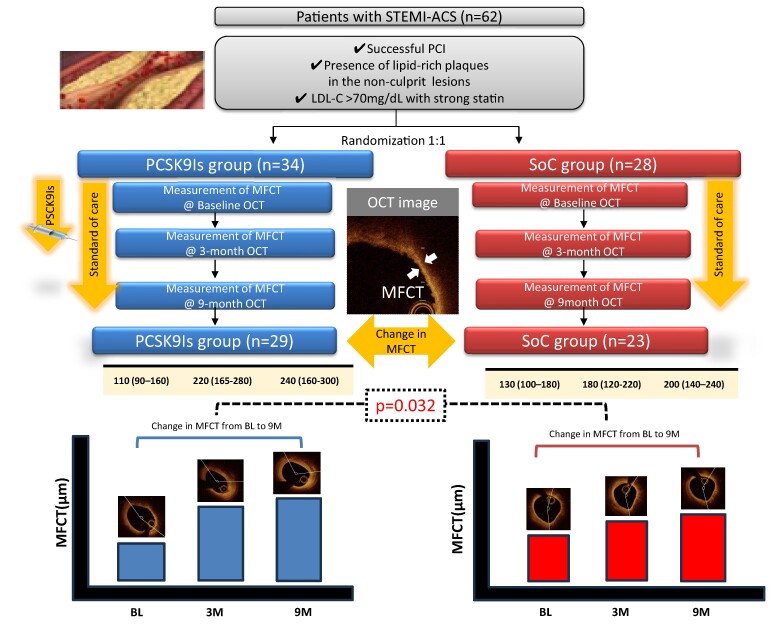
We evaluated the effects of the short-term use of PCSK9Is on coronary plaque stability in patients with ACS using OCT. Participants were randomly assigned to the PCSK9I group (evolocumab 420 mg for 3 months) or standard of care group. Optical coherence tomography was performed at baseline and 3 and 9 months after randomization to assess lipid-rich plaques in non-culprit lesions. Change in the MFCT from baseline to 9 months was the primary endpoint. ACS, acute coronary syndrome; MFCT, minimum fibrous cap thickness; OCT, optical coherence tomography; PCI, percutaneous coronary intervention; PCSK9Is, proprotein convertase anti-subtilisin–kexin type 9 inhibitors; SoC, standard of care; STEMI, ST-elevation myocardial infarction.

## Introduction

Intensive lipid-lowering therapy with statins has been shown to improve outcomes in acute coronary syndrome (ACS).^1^ Some studies have shown that the addition of proprotein convertase anti-subtilisin–kexin type 9 inhibitors (PCSK9Is) to statin therapy further reduces LDL-cholesterol (LDL-C) levels, atheroma volumes, and atherosclerotic cardiovascular events.^2–4^ The HUYGENS study showed that evolocumab administration within 7 days of the onset of non-ST–elevated ACS for 52 weeks resulted in plaque stabilization and regression.^5^ Moreover, the PACMAN-AMI study revealed that initiation of PCSK9Is less than 24 h from ACS onset decreases coronary plaque 52 weeks later.^6^ These studies revealed the benefit of long-term PCSK9I use. A clinical trial is ongoing to access the clinical outcome of PCSK9I administration in ACS (EVOLVE-MI study, AMUNDSEN trial).

A high incidence of cardiovascular events derived from non-culprit lesions has been reported during the first year after ACS^7^ and in even earlier stages of ACS (weeks to months).^8^ Considering that plaque instability and progression also may contribute to these events, the administration of PCSK9Is at an early stage, which facilitates rapid and intensive lipid-lowering, could lead to coronary plaque stabilization and potentially improve long-term outcomes. Current evidence underscores the necessity of long-term administration of PCSK9Is for lipid management to reduce cardiovascular events.^9^ Nevertheless, their high cost and the patient aversion to injectable treatments necessitate a more nuanced approach to their administration. Acknowledging the economic and psychological barriers faced by patients, it is hypothesized that a shorter duration of PCSK9I therapy post-ACS could alleviate financial and injection-related burdens while capturing the initial benefits of plaque stabilization. This study aims to explore the feasibility and efficacy of initiating PCSK9I therapy early after ACS and discontinuing after a brief period (3 months), assessing whether this limited intervention can lead to sustained plaque stability, with the goal of establishing a cost-effective and patient-friendly regimen that does not compromise long-term outcomes, to be evaluated at 9 months in achieving plaque stabilization.

## Methods

### Study design

The study design for the effect of short-term PCSK9I administration on coronary plaque stability in Japanese and Okinawan patients with ACS by optical coherence tomography (OCT) analysis (Adage-Joto study, study registration number: UMIN ID No. 26516) was a multicentre, open-label randomized controlled trial.

### Endpoints

The primary endpoint was change in the minimum fibrous cap thickness (MFCT) from baseline (BL) to the 9-month follow-up. Secondary endpoints included the percentage change in MFCT and lipid arc (LA) from BL to 3- and 9-month follow-up, the change in lumen area, and the angulation of macrophage accumulation (MA) from BL to 3- and 9-month follow-up.

### Study setting

This study was conducted at five sites in Japan (Urasoe General Hospital, Tenyoukai Central Hospital, Naha City Hospital, Yuai Medical Center Hospital, and Kochi Medical Center Hospital), and participants were enrolled from March 2017 to March 2019.

### Ethics

The study was conducted in accordance with the ethical principles outlined in the Declaration of Helsinki and International Conference of Harmonization Guidelines for Good Clinical Practice and all applicable laws, regulations, and rules. This study was approved by the review board of Urasoe General Hospital (approval number 2016A029), and all patients provided written informed consent before enrolment.

### Outcome measures

The OCT examination focused on intermediate stenoses with lipid-rich components unrelated to the culprit lesions in patients with ACS. Optical coherence tomography scans were conducted at the initial evaluation and 3 and 9 months after the initial evaluation. Patients were given a 4-week period to reschedule their examinations at their convenience.

### Data collection

Clinical follow-up visits were scheduled monthly for up to 9 months for routine examination and assessment of adverse events. Mandatory blood lipid profile testing was conducted using blood samples obtained at the initial evaluation and 3 and 9 months after the initial evaluation. Blood samples for the measurement of inflammatory markers were collected according to this schedule in hospitals where this was feasible.

### Patient population

In this study, we identified 80 consecutive cases of patients diagnosed with ST-elevation myocardial infarction (STEMI) who had undergone successful percutaneous coronary intervention (PCI) for the culprit lesion and exhibited mild to moderate stenosis in the non-culprit lesions via angiography. Additionally, these sites were confirmed to have lipid-rich plaques by OCT assessment. The target lesion could be located either in a coronary artery that had undergone PCI or one that had not. Among these, 62 patients, who had started the maximum tolerated dose of atorvastatin (20 mg/day) as per Japanese local guidelines immediately upon hospitalization but did not achieve LDL-C levels below 70 mg/dL before randomization, consented to participate in the trial. These patients were randomly assigned in a 1:1 ratio. Ultimately, serial OCT follow-up data at 3 and 9 months were available for 52 patients, with 29 patients in the PCSK9I group and 23 in the standard of care (SoC) group, all of whom were registered for this trial. The exclusion criteria included cardiogenic shock, the need for coronary artery bypass grafting, renal insufficiency (estimated glomerular filtration rate < 30 mL/min/1.73 m^2^), malignancy, statin intolerance, prior or current use of PCSK9Is, and active systemic inflammation.

### Randomization and masking

All the recruited patients were randomly assigned in a 1:1 ratio to the PCSK9I or the SoC groups by a physician in charge of each facility. Stratified randomization was performed using a web-based allocation system (MUJINWARI SOFTWARE; IRUKA System, Tokyo, Japan), and patients were stratified according to sex, age, diabetes, and participating hospital at BL. Patients in the PCSK9I group took statins plus PCSK9Is (subcutaneous injection of evolocumab 140 mg/2 weeks or 420 mg/month) during hospitalization (median of 9 days) and continued treatment for 3 months after randomization by a physician in charge of each facility. Optical coherence tomography was measured at each time point (BL and 3- and 9-month follow-ups) in both groups. The allocated treatment was not blinded to the patients or investigators but was masked to those involved in the OCT image analysis by removing patient information.

### Optical coherence tomography image acquisition

Optical coherence tomography is a catheter-based imaging method that uses coherent near-infrared light and optical backscatter to produce detailed images of the intimal layers of the coronary artery wall. Consequently, OCT enables a detailed view of the fibrous cap that covers the lipid pools and accurate measurements of its thickness and the LA, which provide the extent of lipid pools indicated as angles. Optical coherence tomography was performed using OPTIS™ IMAGING SYSTEMS (Abbott Cardiovascular, Plymouth, MN, USA). The DRAGONFLY OPSTAR IMAGING CATHETER (Abbott) was calibrated and guided to the target lesion using a 0.35 mm guidewire. The catheter was then placed in the distal part of target lesion, and the contrast medium was injected through the guiding catheter at a rate of 2.5–4 mL/s for a total of 6–12 mL using an injector pump to remove red blood cells. A blood-free image was obtained, and the OCT imaging core was pulled back across the entire target lesion at a speed of 36 mm/s using an automated pullback device. The OCT images were digitally saved for subsequent analyses.

### Optical coherence tomography image analysis

The OCT images were evaluated in a blinded manner using a specialized offline review system (Abbott) at the core laboratory (Urasoe General Hospital Cardiovascular Center). Serial OCT images from the initial evaluation and those taken 3 and 9 months later were compared side by side, and the region of interest (ROI) at each time point was identified by measuring the distance from landmarks including calcifications, branches, and stents. Calibration was performed prior to OCT analysis. The target lesion was required to be located >10 mm away from the PCI-treated lesion in the former case. Plaque characterization was performed using previously validated criteria,^9^ which involved recognizing the lipid core as a diffusely bordered and signal-poor area and the fibrous cap as a signal-rich band covering the lipid core. Macrophage accumulations were defined as the presence of signal-rich, distinct, or confluent punctate regions that exceeded the intensity of background speckle noise. The MFCT determined by examining all consecutive frames (0.2 mm interval/1 frame) could be observed by OCT, choosing lipid-rich plaques (LA > 45 degrees), and calculating the fibrous cap thickness at the thinnest part of the fibrous cap in all the frames observed through OCT. The LA and the angulation of MA were measured within the ROI where the MFCT is present. The inter- and intra-observer reliabilities of this method were evaluated in 30 randomly selected plaques in other OCT images at the core laboratory of Urasoe General Hospital prior to this study. The intra-class correlation coefficient (ICC) for the repeated measurements of fibrous cap thickness by the same observer was excellent [ICC (1,1) = 0.982], with an absolute difference of 15 ± 9 μm. The ICC for measuring fibrous cap thickness by two different observers was also excellent [ICC (2,1) = 0.946], with an absolute difference of 32 ± 13 μm.

### Statistical analysis

In the absence of existing studies on the impact of PCSK9Is on change in MFCT, a moderate effect size of Cohen’s *d* = 0.5 was assumed to design a comparative study with a 20 mg dose of atorvastatin. A pooled standard deviation of 63 μm, derived from prior atorvastatin research,^10^ facilitated the estimation of a mean difference of 31.5 μm. Utilizing standard statistical formulas for a desired power of 80% and a two-sided alpha level of 0.05, the necessary sample size was calculated to be approximately 32 participants per group. Considering a dropout rate of 20%, we estimate a total of 80 cases (40 in the PCSK9I group and 40 in the SoC group). Statistical analyses were performed using JMP (v15.0.0; SAS Institute, Cary, NC, USA). Categorical variables were expressed as frequencies and compared using the *χ^2^* test or Fisher’s exact test (when the expected cell values were <5). Continuous variables were expressed as medians and interquartile range (IQR) and compared using the Mann–Whitney *U* test (for between-group comparison) or Wilcoxon signed-rank test (for longitudinal data analysis). Statistical significance was set at *P* < 0.05. The full analysis set (FAS) included patients who received the allocated treatment and serial OCT examinations [BL and 3 and 9 months] and provided planned and assessable outcome data. The safety analysis set (SAS) included patients who received the allocated treatment at least once. The FAS was used to assess the primary and secondary endpoints. The SAS was used to assess the safety outcomes.

## Results

### Characteristics of the study population

**Figure 1:**
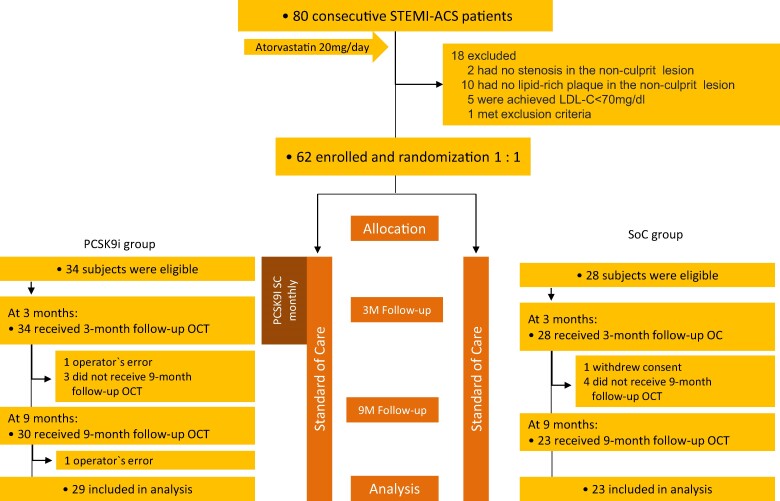
Study profile. Eighty subjects met the inclusion criteria. Of these, 62 participants were randomized at a 1:1 ratio to receive proprotein convertase anti-subtilisin–kexin type 9 inhibitor therapy or standard care. Of these, eight individuals who did not undergo serial optical coherence tomography examinations at baseline and 3 and 9 months were excluded. Ultimately, 52 patients underwent serial optical coherence tomography examinations in accordance with the protocol, with 29 in the proprotein convertase anti-subtilisin–kexin type 9 inhibitor group and 23 in the standard of care group.

The study population comprised 62 patients (62 lesions), as depicted in *Figure 1*. Participants were randomized in a 1:1 ratio to receive PCSK9I therapy or standard care. Of the 62 individuals, 10 were excluded from the study: 7 dropped out of the OCT examinations, 2 did not obtain appropriate OCT images due to operator error, and 1 withdrew consent. Ultimately, 52 patients (52 lesions) underwent serial OCT examinations in accordance with the protocol, with 29 in the PCSK9I group and 23 in the SoC group, respectively. The median duration from the onset of ACS to the initiation of PCSK9I therapy was 9 days (IQR: 7–13 days). Baseline characteristics and concomitant medications of the two groups are presented in Supplementary material online, *Tables S1* and *S2*, respectively. Supplementary material online, *Table S3*, shows the doses of lipid-lowering drugs over time.

### Changes in biochemical measurements

Serum total cholesterol, LDL-C, HDL-cholesterol (HDL-C), triglycerides, haemoglobin A1c, high-sensitivity C-reactive protein, lipoprotein(a), and malondialdehyde-modified LDL (MDA-LDL) levels were similar between the two groups at BL (see Supplementary material online, *Table S3*). Significant reductions in LDL-C compared with BL levels were observed in the PCSK9I and the SoC groups at 3 and 9 months after the initiation of the study, and the reduction in the PCSK9I group was significantly greater than that in the SoC group at 3 months but not at 9 months; however, the difference disappeared 6 months after PCSK9I discontinuation, specifically at 9 M after the initiation of the study. No apparent effects of PCSK9I were observed on haemoglobin A1c or triglyceride levels.

### Primary and secondary endpoints

**Figure 2:**
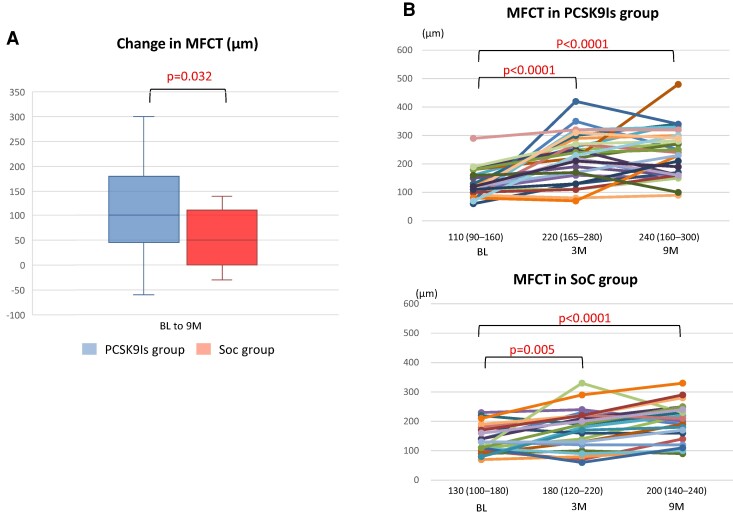
Change in the minimum fibrous cap thickness by optical coherence tomography measurement. (*A*) Change in the minimum fibrous cap thickness from baseline to the 9-month follow-up was significantly greater in the proprotein convertase anti-subtilisin–kexin type 9 inhibitor group than in the standard of care group. (*B*) Minimum fibrous cap thickness was significantly greater at 3 and 9 months than at baseline in the proprotein convertase anti-subtilisin–kexin type 9 inhibitor and the standard of care groups.

**Figure 3:**
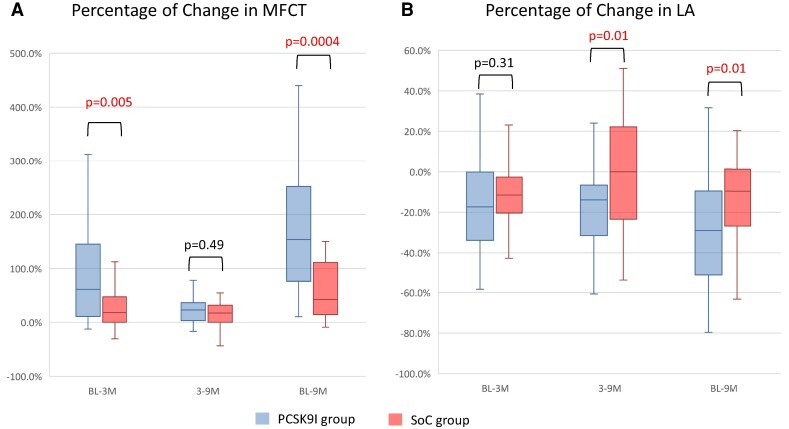
The percentage change in optical coherence tomography measurements. (*A*) The percentage change in the maximum fibrous cap thickness from baseline to the 3-month follow-up and from baseline to the 9-month follow-up was significantly greater in the proprotein convertase anti-subtilisin–kexin type 9 inhibitor group than in the standard of care group. (*B*) The percentage change in maximum lipid arc from 3 to 9 months and from baseline to 9 months was significantly reduced.

**Figure 4:**
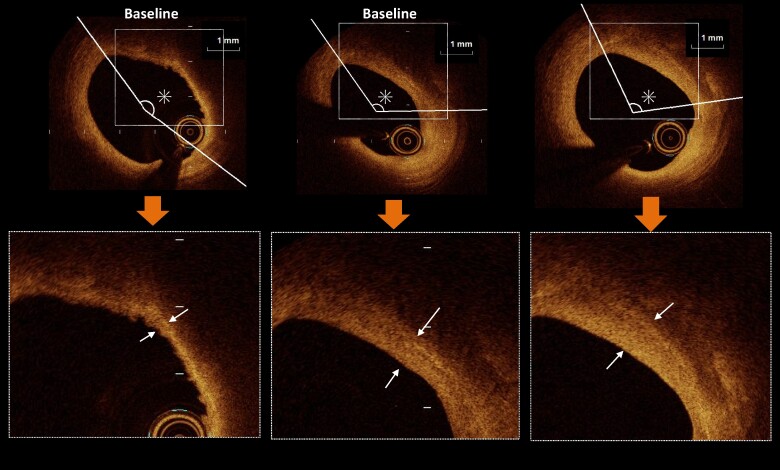
Representative optical coherence tomography images in the proprotein convertase anti-subtilisin–kexin type 9 inhibitor group. Fibrous cap thicknesses (white arrows) were increased from baseline (80 mm) to the 3-month follow-up (350 mm) and from the 3-month follow-up to the 9-month follow-up (500 mm); lipid (asterisk) arcs were decreased during the follow-up period.

**Figure 5:**
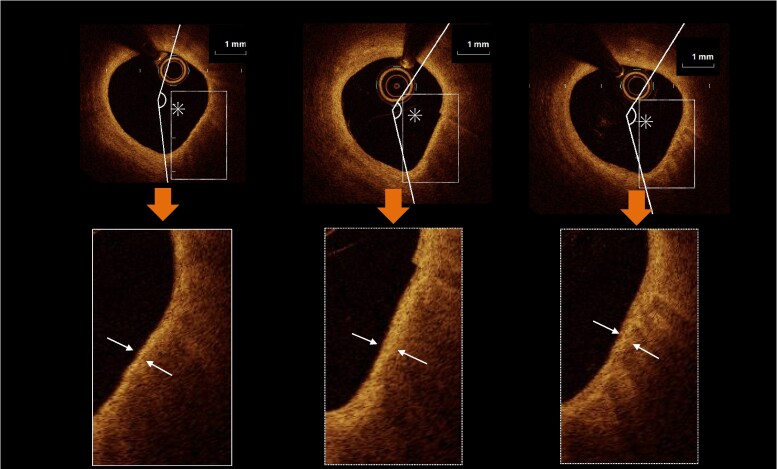
Representative optical coherence tomography images in the standard of care group. Fibrous cap thicknesses (white arrows) were mildly increased from baseline (80 mm) to the 3-month follow-up (140 mm) and from the 3-month follow-up to the 9-month follow-up (220 mm); lipid (asterisk) arcs were slightly decreased during the follow-up period.

*Figure 2A* and *B* show the change in MFCT at 9 months from BL, with individual data represented at every time point of the OCT measurements. Changes in MFCT from the BL were significantly greater in the PCSK9I group than in the SoC group at 9 months [100 μm (IQR: 45–180 μm) vs. 50 μm (IQR: 0–110 μm) at BL to the 9-month follow-up, *P* = 0.032]. *Figure 3* shows the percentage changes in the MFCT and LA. The MFCT was significantly increased from BL to the 3-month and 9-month follow-ups in the PCSK9I group compared with the SoC group [37.5% (IQR: 4.7–122.7%) vs. 12.7% (IQR: 0–42.9%) at BL to 3 months, *P* = 0.012; 54.2% (IQR: 5.0–166.7%) vs. 33.2% (IQR: 0.0–76.6%) at BL to 9 months, *P* = 0.029; *Figure 3A*]. Lipid arc was significantly decreased from BL to 9 months in the PCSK9I group compared with the SoC group [−21.6% (IQR: −39.9–0.0%) vs. −1.8% (IQR: −12.7–1.5%), *P* = 0.02; *Figure 3B*]. *Table 1* shows the results of the OCT analysis in the PCSK9I and the SoC groups, i.e. MFCT and LA at BL and 3 and 9 months after the commencement of this trial. The MFCT significantly increased and LA, and the angulation of MA significantly decreased at 3 and 9 months compared with the BL values in both groups. Regarding between-group comparisons, MFCT was significantly higher in the PCSK9I group than in the SoC group at 3 and 9 months. The LA and the angulation of MA were lower in the PCSK9I group than in the SoC group at 9 months. *Figures 4* and *5* show representative OCT images in the PCSK9I and the SoC groups.

**Table 1:** Optical coherence tomography measurements

Measurement	PCSK9i group (*n* = 29)			SoC group (*n* = 23)		
	Baseline [median (IQR)]	3 months [median (IQR)]	9 months [median (IQR)]	Baseline [median (IQR)]	3 months [median (IQR)]	9 months [median (IQR)]
MFCT (µm)	110 (90–160)	220 (165–280)**	240 (160–300)**	130 (100–180)	180 (120–220)*	200 (140–240)**
Lipid arc with MFCT (degree)	128 (97–155)	100 (81–130)*	93 (74–113)**	136 (96–147)	118 (82–147)	112 (76–133)*
Lumen area with MFCT (mm²)	6.9 (4.7–7.9)	5.7 (4.5–7.6)	6.9 (5.5–8.1)	6.3 (4.5–7.8)	5.9 (4.1–8.2)	6.3 (3.6–8.5)
The angulation of macrophage accumulation with MFCT (degree)	41.9 (28.7–65.0)	30.8 (16.8–42.8)*	12.2 (0.0–29.2)**	47.9 (21.8–68.4)	41.2 (0.0–58.6)	48.0 (15.9–62.3)

Values are median (IQR).

IQR, interquartile range; MFCT, minimum fibrous cap thickness; PCSK9i, proprotein convertase anti-subtilisin–kexin type 9 inhibitor; SoC, standard of care.

^*^ *P* < 0.05 vs. baseline.

^**^ *P* < 0.05 vs. SoC group.

### Safety and clinical outcomes

There was one case of target lesion revascularization after the initiation of the trial in each group [one (3.4%) in the PCSK9I group vs. one (4.3%) in the SoC group]. However, no cardiac deaths, target lesion–related myocardial infarctions, or adverse drug reactions occurred in either group.

## Discussion

In this randomized controlled trial, we showed that the increase in MFCT with PCSK9I treatment in the first 3 months after ACS was maintained for 6 months after treatment discontinuation, although the effect of PCSK9I on LDL-C disappeared after discontinuation. These results are consistent with those of the study by Nicholls *et al*.^5^ that demonstrated the favourable effects of statin and PCSK9I combination on plaque stabilization and regression. However, our findings provide additional clinically important evidence for the mid-term benefit of intensive LDL-C–lowering therapy with statins and PCSK9Is, even for a relatively short duration, on plaque stabilization and regression.

### The explanation for a more stabilized plaque

The CLIMA study revealed that OCT-high-risk coronary plaques with macrophage clusters, indicating plaque inflammation, were more prevalent in patients who experienced coronary events such as ACS.^11^ Peri-coronary adipose tissue attenuation on coronary computed tomography angiography is significantly higher in patients with ACS than in those with chronic coronary syndrome, possibly indicating a more intense inflammatory response.^10,12^ Furthermore, ACS triggers an inflammatory response by releasing cytokines,^12,13^ which can destabilize plaques in non-culprit lesions and cause recurrent cardiac events in a relatively short duration. The stabilization of plaques during combination treatment with PCSK9Is and statins may be attributed to a substantial decrease in LDL-C and direct anti-inflammatory effects. Plasma PCSK9 levels are elevated after an ACS event,^14^ which may augment inflammation, even without the hyperlipidaemic effect. The PCSK9 triggers the expression of endothelial adhesion molecules and nuclear translocation of NF-κB, resulting in increased messenger ribonucleic acid levels and secretion of pro-inflammatory cytokines, such as tumor necrosis factor alpha, interleukin (IL)-1β and IL-6, and decreased levels of anti-inflammatory cytokines, such as IL-10 and arginase.^15–19^ Recent findings indicate that the administration of PCSK9Is suppresses the expression of inflammatory markers and stabilizes human atherosclerotic plaques.^20^ Another study demonstrates that increased macrophage content within plaques correlates with heightened inflammatory responses and is indicative of plaque vulnerability, suggesting a link between MA angles and inflammation intensity in coronary arteries.^21^ The PCSK9 decreases cholesterol efflux via ATP Binding cassette A1 (ABCA-1) by reducing ABCA-1 gene and ABCA-1 protein expression.^22^ The PCSK9 plays a role in foam cell formation by activating scavenger receptors and suppressing ABCA1 receptors, potentially decreasing plaque removal capacity. Therefore, the administration of PCSK9Is not only offers intensive lipid-lowering effects but also may stabilize plaques following ACS through these multifaceted effects.

### Clinical implications of the results

Several clinical trials have shown that long-term combination treatment with PCSK9Is and statins significantly lowers LDL-C levels and reduces the risk of cardiovascular events,^2,3^ presumably through atheroma regression.^4^ A recent Fourier OLE trial showed the benefits of the long-term use of this combination in high-risk patients.^20^ However, the long-term use of PCSK9Is is much more burdensome for patients than statins alone, in terms of drug costs, cumbersome of injectable device, and frequent hospital visits. Although there is no dispute about the usefulness of long-term administration, no studies have evaluated whether the early initiation and short-term use of PCSK9Is in the acute phase of ACS can regress coronary plaques in the chronic phase after PCSK9I discontinuation. Patients in the immediate aftermath of an ACS event are ideal candidates for this treatment since the risk of recurrent coronary events is highest in the few weeks to months following the event. Vulnerable plaques after an ACS event may form not only in the non-culprit lesion but also throughout the coronary arteries.^21^ Consequently, a plan for plaque stabilization in non-culprit lesions must be implemented after successful PCI. We demonstrated that the use of PCSK9Is with statins for 3 months post-ACS was more successful in stabilizing plaques than statin treatment alone. We also indicated that the plaque-stabilizing effect persisted for 6 months after PCSK9I discontinuation, and LDL-C levels in the SoC and PCSK9I groups were similar at this time point. The exact mechanism of this ‘sustained effect’ is unclear; however, it is believed that plaque stabilization was attained during the period of high risk of relapse after a relatively brief course of treatment, which reduced the patient’s burden. Inflammation is a critical factor in the vulnerability and progression of atherosclerotic plaques. Consequently, the anti-inflammatory properties of PCSK9Is could play a significant role in plaque stabilization. Given that inflammation intensifies immediately following ACS, the early administration of PCSK9Is post-ACS may enhance the stabilization of plaques. Moreover, even short-term administration during periods of heightened plaque progression risk could potentially achieve plaque stabilization. Recent guidelines suggest that it may be more beneficial to initiate PCSK9I/statin combinations in an earlier stage of ACS during high-risk periods and in high-risk patients.^23^ One study has shown that even short-term treatment with PCSK9Is (for 6 months) resulted in markedly decreased LDL levels and had a positive effect on the risk of cardiovascular events that persisted beyond the treatment period (sustained effect) in patients with ACS,^24^ and a retrospective study reported that the early initiation and short-term (3 months) use of PCSK9Is significantly reduced the composite endpoints at 1 year,^25^ which supports the findings of our study. Future studies with extended follow-up periods are necessary to fully ascertain the duration and stability of the sustained effect of PCSK9Is in plaque stabilization. This will help in better understanding the long-term cardiovascular benefits and cost-effectiveness of short-term PCSK9I therapy in the management of ACS.

### Limitations

This study had several limitations. First, we enrolled potentially selected populations, that is, those who underwent successful primary PCI and had LDL-C levels > 70 mg/dL while receiving high-intensity statin therapy. Moreover, we only analysed the efficacy per-protocol population, that is, those who received each allocated treatment during the study period and completed the serial OCT examinations. Second, there is no scientific rationale for the duration of PCSK9I treatment after ACS and OCT performance at the 3-month follow-up. Third, OCT is partly subjective, although we attempted to maintain objectivity in determining MFCT using the ICC. Fourth, if the ROIs were selected in the infarct-related vessel, the target lesions were required at least 10 mm away from the culprit lesions to avoid the effects of catheter intervention such as stenting or balloon injury. However, the possibility of plaque progression due to catheter-related mechanical irritation might be remained. Fifth, all patients received atorvastatin at 20 mg/day, which is the maximum approved dose in Japan but is classified as moderate-intensity therapy in the USA and Europe. The effect of PCSK9Is on LDL-C and plaque stabilization might have been attenuated if higher doses of statins were used. Finally, our clinical trial was conducted in a small number of patients since it aimed to assess plaque stabilization by OCT, and recurrence after ACS was not assessed. Moreover, as this study evaluated the mid-term effects on the stabilization of coronary plaque, a large-scale clinical trial investigating the long-term effects is necessary. Our results may provide a rationale for conducting trials to evaluate the effects of short-term concomitant administration of PCSK9Is and statins after ACS on cardiovascular outcomes.

## Conclusions

Aggressive LDL-C lowering with a combination treatment of PCSK9Is and statins after ACS resulted in more marked plaque stabilization than the SoC alone, and this effect persisted for up to 6 months, even after PCSK9I discontinuation.

## Lead author biography


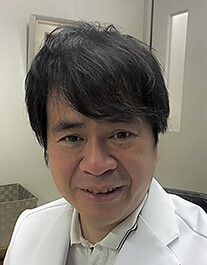


Hiroki Uehara, M.D. I am currently serving as the chief of the Department of Cardiology at Urasoe General Hospital and am a council member of the Kyushu-Okinawa branch of the Japanese Society of Cardiovascular Intervention and Therapeutics (CVIT). I hold certification in catheter treatment and have extensive experience in interventional cardiology. My research interests focus on the treatment of complex lesions, cardiovascular imaging, and pharmacological interventions for atherosclerosis. I am committed to advancing cardiovascular care through innovative therapies and a patient-centred approach.

## Supplementary Material

oeae055_Supplementary_Data

## Data Availability

The data underlying this article will be shared on reasonable request to the corresponding author.
