# Supplementary material for: Early and short-term use of proprotein convertase anti-subtilisin–kexin type 9 inhibitors on coronary plaque stability in acute coronary syndrome
Source: Eur Heart J Open. 2024 Jul 23;4(4):oeae055. doi: 10.1093/ehjopen/oeae055 (PMC11316204; doi:10.1093/ehjopen/oeae055)
Supplement: oeae055_Supplementary_Data [file oeae055_supplementary_data.docx]

**Supplementary material online**

**Early and Short-term Use of PCSK9-Inhibitors on Coronary Plaque Stability in Acute Coronary Syndrome** **(Adage-Joto study)**

**Hiroki Uehara, MD^a,b*^, Takashi Kajiya, MD^c^, Masami Abe, MD^d^, Marohito Nakata, MD^e^, Shingo Hosogi, MD, PhD^f^, Shinichiro Ueda MD, PhD^b^**

^a^Department of Cardiology, Urasoe General Hospital, Okinawa, Japan

^b^Department of Clinical Research Education and Management, University of Ryukyus Graduate School of Medicine, Okinawa, Japan

^c^Department of Cardiology, Tenyoukai Central Hospital, Kagoshima, Japan

^d^Department of Cardiology, Yuai Medical Center Hospital, Okinawa, Japan

^e^Department of Cardiology, Naha City Hospital, Okinawa, Japan

^f^Department of Cardiology, Kochi Medical Center, Kochi, Japan

Table of contents

[Table S1. Baseline characteristics 2](#_Toc157940372)

[Table S2. Concomitant medications 2](#_Toc157940373)

[Table S3. Lipid-lowering agents 3](#_Toc157940374)

[Table S4. Blood sample data 3](#_Toc157940375)

# Table S1. Baseline characteristics

| **Characteristics** | **PCSK9Is group**  **(n=29)** | **Soc group**  **(n=23)** | **P Value** |
| --- | --- | --- | --- |
| Age (years) | 59.6 (11.9) | 60.0 (12.9) | 0.75 |
| Men, *n* (%) | 22 (75.8) | 16 (69.5) | 0.61 |
| Hypertension, *n* (%) | 16 (55.1) | 14 (60.8) | 0.78 |
| statin naïve, *n* (%) | 23 (79.3) | 21 (91.3) | 0.22 |
| Diabetes mellitus, *n* (%) | 10 (34.4) | 8 (34.7) | 0.98 |
| Current smoking, *n* (%) | 13 (44.8) | 13 (56.5) | 0.54 |
| Family history of CAD, *n* (%) | 3 (10.3) | 4 (17.3) | 0.52 |
| Target vessel |  |  | 0.44 |
| LAD, *n* (%) | 15 (51.7) | 13 (56.5) | - |
| LCx, *n* (%) | 4 (13.7) | 5 (21.7) | - |
| RCA, *n* (%) | 10 (34.4) | 5 (21.7) | - |
| Location of target plaque |  |  | 0.93 |
| Culprit vessel, *n* (%) | 25 (86.2) | 20 (86.9) | - |
| Nonculprit vessel, *n* (%) | 4 (13.7) | 5 (21.7) | - |
| Data are presented as mean ± standard deviation unless stated otherwise. N. S = not significant; CAD = coronary artery disease; LAD = left anterior descending coronary artery; LCx = left circumflex coronary artery; RCA = right coronary artery | | | |

# Table S2. Concomitant medications

|  | **PCSK9I group (n=29)** | **SoC group (n=23)** | **P Value** |
| --- | --- | --- | --- |
| Aspirin, *n* (%) | 29 (100) | 23 (100) | N.S |
| P2Y12R inhibitors, *n* (%) | 29 (100) | 23 (100) | N.S |
| Beta blockers, *n* (%) | 20 (71) | 19 (82) | 0.34 |
| ACE inhibitors or ARBs | 17 (60) | 17 (73) | 0.31 |
| Oral hypoglycemic agents, *n* (%) | 9 (32) | 4 (17) | 0.22 |
| Insulin, *n* (%) | 2 (7) | 0 (0) | 0.11 |
| Ezetimib, *n* (%) | 2 (7) | 6 (26) | 0.054 |
| N. S = not significant, P2Y12R = platelet P2Y12 receptor, ACE = Angiotensin-converting enzyme  ARBs = Angiotensin receptor blockers | | | |

# Table S3. Lipid-lowering agents

|  | **PCSK9I group (n=29)** | | | **SoC group (n=23)** | | |
| --- | --- | --- | --- | --- | --- | --- |
|  | **Baseline** | **3-month** | **9-month** | **Baseline** | **3-month** | **9-month** |
| Atrorvastatin 20mg, *n* (%) | 29 (100) | 29 (100) | 29 (100) | 23 (100) | 23 (100) | 23 (100) |
| Ezetimib 10mg, *n* (%) | 0 (0) | 0 (0) | 2 (7) | 2 (8) | 6 (26) | 6 (26) |
| There are no significant changes between the two groups. | | | | | | |

# Table S4. Blood sample data

|  | **PCSK9I group (n=29)** | | | **SoC group (n=23)** | | |
| --- | --- | --- | --- | --- | --- | --- |
|  | **Baseline** | **3-month** | **9-month** | **Baseline** | **3-month** | **9-month** |
| TC, mg/dL | 156 (136–198) | 106 (80–115)*† | 149 (124–156) | 140 (134–164) | 125 (116–164) | 156 (126–169) |
| LDL-C, mg/dL | 102 (78–119) | 25 (16–42)*† | 70 (63–90)* | 92 (80–114) | 79 (61–90)* | 80 (67–93)* |
| HDL-C, mg/dL | 38 (33–44) | 47 (41–59)*† | 44 (38–49)* | 38 (34–43) | 42 (39–51)* | 49 (44–55)* |
| TG, mg/dL | 126 (89–159) | 122 (76–169) | 106 (83–138) | 118 (95–134) | 107 (82–158) | 107 (88–138) |
| HbA1c, % | 6 (5.6–7.2) | 6.1 (5.8–6.9) | 6.2 (5.7–6.6) | 6 (5.6–6.7) | 6.1 (5.8–6.5) | 6 (5.7–6.6) |
| hsCRP^‡^, mg/dL | 0.4 (0.26–1.18) | 0.06 (0.03–0.17)* | 0.05 (0.03–0.09)* | 0.49 (0.26–1.13) | 0.13 (0.08–0.43)* | 0.08 (0.05–0.08)* |
| Lp(a) ^‡ ‡^, mg/dL | 18 (15–37) | 8.5 (3–21) | 12 (7–28)* | 17 (11–33) | 10 (5–25)* | 10 (5–24)* |
| MDA-LDL^‡ ‡ ‡^, U/L | 99 (70–127) | 69 (46–92)*† | 106 (68–108) | 95 (76–123) | 83 (67–89) | 91 (62–111) |
| Values are median (interquartile range). *p < 0.05 versus baseline. †p < 0.05 versus SoC group.  HbA1c = hemoglobin A1c; HDL-C = high-density lipoprotein cholesterol; LDL-C = low-density lipoprotein cholesterol; TC = total cholesterol; TG = triglyceride; hs CRP = highly sensitive C reactive protein ; Lp(a) = Lipoprotein a; MDA-LDL = malondialdehyde-modified low-density lipoprotein.  ^‡^ n=10 (PCSK9I group) versus n=10 (SoC group). ^‡ ‡^ n=18 (PCSK9I group) versus n=16 (SoC group). ^‡ ‡ ‡^ n=9 (PCSK9I group) versus n=8 (SoC group). | | | | | | |
